# Evolution of the ocular immune system

**DOI:** 10.1038/s41433-024-03512-4

**Published:** 2024-12-09

**Authors:** John V. Forrester, Paul G. McMenamin

**Affiliations:** 1 University of Aberdeen, School of Medicine, Medical Sciences and Nutrition, Institute of Medical Sciences, Foresterhill, Aberdeen, AB25 2ZD Scotland UK; 2 Emeritus Professor, Monash University, Clayton, VIC Australia

**Keywords:** Autoimmunity, Epidemiology

## Abstract

The evolution of the ocular immune system should be viewed within the context of the evolution of the immune system, and indeed organisms, as a whole. Since the earliest time, the most primitive responses of single cell organisms involved molecules such as anti-microbial peptides and behaviours such as phagocytosis. Innate immunity took shape ~2.5 billion years ago while adaptive immunity and antigen specificity appeared with vertebrate evolution ~ 500 million years ago. The invention of the microscope and the germ theory of disease precipitated debate on cellular versus humoral immunity, resolved by the discovery of B and T cells. Most recently, our understanding of the microbiome and consideration of the host existing symbiotically with trillions of microbial genes (the holobiont), suggests that the immune system is a sensor of homoeostasis rather than simply a responder to pathogens. Each tissue type in multicellular organisms, such as vertebrates, has a customised response to immune challenge, with powerful reactions most evident in barrier tissues such as the skin and gut mucosa, while the eye and brain occupy the opposite extreme where responses are attenuated. The experimental background which historically led to the concept of immune privilege is discussed in this review; however, we propose that the ocular immune response should not be viewed as unique but simply an example of how the tissues variably respond in nature, more or less to the same challenge (or danger).

## Introduction

The notion that there is a distinct ocular immune system assumes that it evolved from an existing, common immune system, which begs the question of how the immune system evolved in the first instance. Our understanding of immunity began with the invention of the microscope, separately by Leeuwenhoek and Hooke [reviewed in ref. 1], the discovery of unicellular live organisms [2], and germ theory as postulated by Koch and Pasteur in the 19th century [3, 4]. Metchnikoff [5] proposed that microbe-phagocytosing cells (leucocytes) were the foot-soldiers of host defence (cellular immunity) which sparked off a prolonged debate with Ehrlich who considered chemical factors (antisera) the basis of host protection (humoral immunity) [6].

The concept of immunity, and its regulation, in eukaryotic multicellular organisms continues to evolve. We now have a modified view of “germs” with our current understanding of the microbiome and the extensive degree to which the host depends on symbiosis / commensalism / mutualism with the microbiome. Indeed, it is now considered that symbiosis/mutualism with microbes probably helped drive the development of the three embryonic layers in multicellular life forms – a process known as gastrulation.

**Fig. 1:**
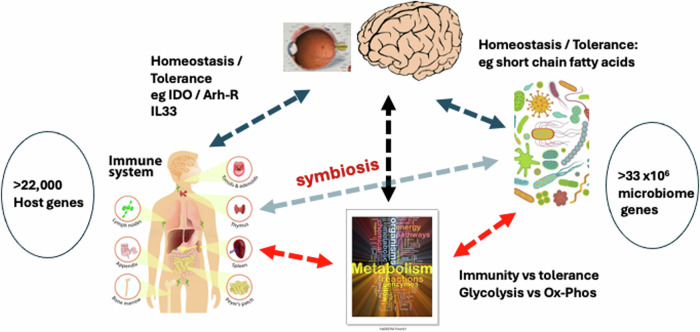
Symbiosis and the Holobiont. Symbiosis and the Holobiont: Host genes plus microbiome genes constitute the holobiont which maintains homoeostasis (immunological tolerance) through symbiosis, mediated by immune regulatory molecules such as indoleamine (IDO), the aryl hydrocarbon receptor (Arh-R), interleukin 33 (IL33) in host cells and short chain fatty acids from the microbiota. The CNS (the eye and the brain) controls homoeostasis by feedforward and feedback interactions through neural and signalling networks which contribute to important regulatory mechanisms such as blood CNS barriers (see later text for details). Host metabolism plays a major role in maintaining homoeostasis: disturbance of homoeostasis is fuelled through an increase in glycolysis while homoeostasis is maintained through oxidative phosphorylation (Ox-Phos). (Figure complied from images provided by istockphoto.com).

The original notion of uni- and multi-cellular eukaryotic organisms defending themselves against prokaryotes has undergone a massive re-think since the “discovery” of the microbiome and the use of whole genome sequencing (WGS) in studies of evolutionary phylogeny. Humans have ~22,000 host genes and >33 million genes in the microbiota, comprising bacterial, fungal, viral and parasite genes, mostly distributed on surfaces exposed to the external elements (skin, gut, airways) [7]. The combined gene pool is contained within what is termed the holobiont [8–10] and many physiological and homoeostatic functions of the holobiont are dependent on co-operative interactions between host genes and microbiome genes. This does not negate the germ theory of disease but opens a new perspective on how “germs” engage in their pathogenic activities. In evolutionary terms, symbiosis between eukaryotes and prokaryotes / archaea probably is much more of a deciding factor on whether a microbe becomes a pathogen or enters a mutualistic state with the host (Fig. 1).

## Evolution of the immune system

**Fig. 2:**
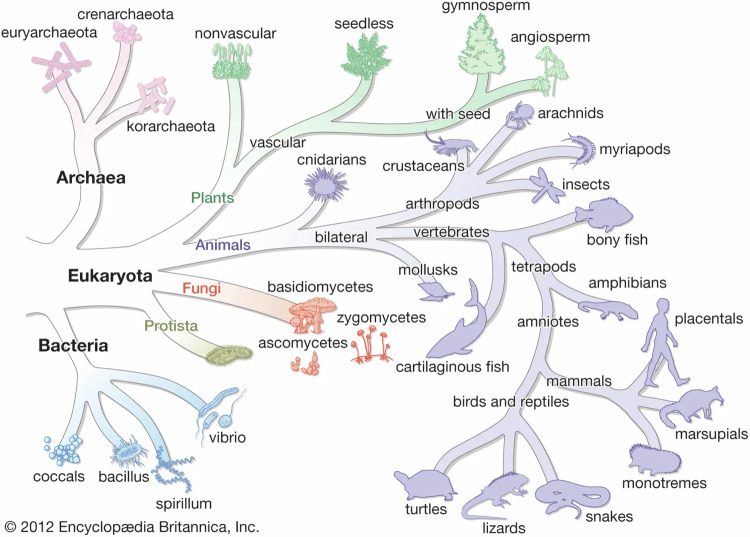
The Tree of Life. Tree of Life indicating estimated timing of appearance of innate and adaptive immunity during evolution. Adaptive immunity coincides with appearance of vertebrates while evidence of innate immunity is present in unicellular organisms. (By courtesy of Encyclopædia Britannica, Inc., copyright 2021; used with permission.).

As Kaufman has said, the evolution of an immune system can be considered on a number of time-scales: evolution of life on earth, evolution over the lifetime of a single species, or the evolution of clones of immune cells after a single antigenic challenge [11]. Evolution of life on earth is considered to begin with the last universal common ancestor (LUCA) and to have diversified to the three domains of life around 3.5–3.8 billion years ago (bya) [12]. Ancient microbicidal molecules such as antimicrobial peptides and co-opted morphogens such as TGFβ and Wnt/Frizzled, co-evolved with unicellular organisms and represent examples of fundamental defence mechanisms which persist through to modern times. Unicellular organisms such as amoeba were seen to have an “ancient system of host defence” based on the evidence of shared genes such as NFκB [13], and plants, protozoans and metazoans are all recognised to have the core machinery of an innate immune system. Innate immunity in the guise of cells emerged around 1.0 bya by which time phyla of multicellular organisms had appeared (Fig. 2). Innate immunity emerged from primitive single cell amoeba-like phagocytic removal of pathogens, to the development of, for instance, Toll-like receptors [13] when bilateria (bilaterally symmetrical organisms) appeared around 700 million years ago (mya) [14]. Adaptive immunity evolved with the appearance of vertebrates (jawed fish) 450 mya [15–18] and was characterised by exquisite antigen specificity mediated through recombinase (RAG) genes directing the generation of T and B cell receptors, and by immunological memory (recall).

Despite convergence and overlap in several functional behaviours, innate and adaptive immunity are recognised as two separate arms of the vertebrate immune system. Innate immunity was considered non-specific and not to have memory, while adaptive immunity exhibited high levels of antigen-specific memory. However, even these pillars of dogma have undergone conceptual evolution. For example, the dichotomy between cellular and humoral immunity was resolved when, first, Jacques Miller described the thymic origin of a subset of lymphocytes (T cells) and shortly thereafter, Max Cooper identified B lymphocytes [from “Bursa of Fabricius” in chickens and their equivalent in mammals [19]]. Since these early studies much else has been discovered such as the unique specificity of antibodies, B and T cell receptors, regulatory T and B cells, and the harnessing of monoclonal antibodies for therapy. Further dogma was revised when a degree of specificity was also discovered to underpin innate immunity. Janeway demonstrated that innate immune cells used pattern recognition receptors (PRRs) to recognise evolutionarily conserved molecules on pathogens in the form of generic patterns (pathogen associated molecular patterns, PAMPs) common to subclasses of microbes, such as bacteria and viruses [13]. Matzinger expanded on these findings to suggest a refined process whereby immune cells responded to a variety of “danger” and “damage” signals by recognising danger / damage associated molecular patterns (DAMPs): this could include anything (PAMPs and DAMPs and more) which induced a change in tonic, basal immune cell activity [20].

In a broader sense, both innate and adaptive immunity in mammals, such as humans, are quite selective in what they consider a challenge; most antigens (either self or microbiome-derived) are “harmless” and although they are “recognised” by the immune system they do not induce an immune response. Induction of a pro-inflammatory immune response requires activation of innate immune receptors by PAMPs/DAMPs, which in the right context (additional costimulatory molecules) leads to antigen-specific adaptive immunity. This process is different for each tissue. In addition, innate immune cells demonstrate some degree of memory, termed “trained immunity,” [21] which has an evolutionary provenance [22]. The microbiome, meanwhile, keeps the immune system in a constant state of basal (tonic) activation described as parainflammation [23, 24] which can be tipped into overt activation by pathogens.

## Immunity, tolerance and the microbiome

Originally conceived as the mechanism whereby multicellular organisms detect and protect themselves from attack by predatory elements, the immune system has come to be viewed more as a “sensor” of homoeostasis. Rather than solely differentiating between “self” and “non-self” as proposed by Burnet [reviewed in ref. 25], or more broadly between “dangerous” and “harmless” agents, as proposed by Matzinger [20, 26], there is a move towards a newer concept of the immune system whereby not only is “change” detected by immune cells and molecules but the critical determinant is speed of change. This is known as the “discontinuity theory” [27, 28]. According to this notion, a sudden change in the environmental ecosystem induces sets of specific signalling events in the resident detector cells (innate immune cells) leading to recruitment of cells and molecules programmed to clear the danger (usually a pathogen) which has initiated the change, and leads to “memory” against future attacks by the same pathogen. This is classical immunity. In contrast, immune cells sensing a gradual change, such as that produced by damaged, dying, or senescent cells (of host or non-host origin), do not induce a marked change in homoeostasis or a prominent immune response, and this is classical immune tolerance [20]. Even chronic infection, especially infections which develop slowly, induce a tissue-selective form of “disease tolerance”, driven by various metabolic pathways, particularly glucose metabolism (Fig. 1) [29–34]. Thus, the immune system is seen as the “guardian” of homoeostasis [35] and is never at rest in its constant interactions with the microbiome. Indeed, the recognition of multiple genera of microbes not only as harmless but as potentially beneficial symbionts has been proposed as a driver not only of immune homoeostasis but of the development and evolution of diversity of life generally [36]. The immune system and the constant microbial interaction with evolving multicellular organisms likely drove the evolution of ancient “host” responses such as antimicrobial peptides and the development of specialised “sensory” cells (including neuronal and immune cells) [35].

In mammals, the developing foetal / neonatal immune system is critically dependent on the maternal microbiome [37]. Once established as a network of cells, resident in the tissues as well as in secondary lymphoid organs, both innate immune cells (such as dendritic cells, macrophages, and mast cells) and adaptive immune cells (such as T cells, B cells, tissue resident lymphocytes) “patrol” or monitor not only external/internal tissue barrier sites (skin, lungs, gut, genitourinary tract) but also non-barrier sites for pathogens, toxins, damaged and aged cells or proteins, and other “threats”. Innate immune cells, with their “danger” recognition machinery, are particularly evident at external barrier interfaces, but also at internal barrier sites. For example, in the retina and brain, microglia, a yolk sac-derived group of CNS-restricted specialised macrophages, contribute to the neurovascular unit (NVU), a tightly-knit complex of cells characteristic of the blood-retina and -brain barriers [38]. Under homoeostatic conditions, most of these “threats” are dealt with without inducing inflammation. However, if homoeostasis is perturbed, the immune system will respond by becoming activated. This may depend on the rate or speed of perturbation [discontinuity theory of immunity, ref. 28], the nature of the challenge (danger theory, DAMPs) or the type of pathogen / toxin (PAMPs) as outlined above. Self-proteins are constantly recognised by immune cells but do not act as DAMPs or PAMPs. Instead, they invoke immune tolerance, are quietly removed by autophagy, and their components re-cycled by tissue phagocytes. In this sense, self-antigens are little different from the myriad host commensal “self” antigens which are normally cleared by the immune system. Thus, not only does the host tolerate the products of its 22,000 “self” genes but  it is also tolerant of the gene products of the >3.3 m non-redundant genes of the microbiome [7] (see Fig. 1 and legend). However, both commensal and self-antigens can become “dangerous” if they become altered and are recognised in a particular context, for example, by an already activated innate immune cell. Altered “self” antigen (autoantigen) [25] can be regarded as an example of a “threat” in line with the PAMP / DAMP system described above, and may activate autoreactive T cells if presented in context with PAMP/DAMP innate immune cell activation, similar to pathogen-derived antigen.

## The immune system in action

Dishaw et.al. stated that “immune systems evolve to maintain homoeostasis with the environment, prevent microbial assault and recycle damaged host tissues” [39]. Under homoeostatic conditions hosts, commensals, danger molecules, and pathogens all co-exist in a state of symbiosis or mutualism (symbionts) as described above. Symbionts can be viewed as competitors within the host ecosystem and the microbiome itself is seen as “an ecosystem on a leash” [40]. When homoeostasis is disrupted, and the immune system is programmed to get rid of a pathogen, or damaged cells and proteins, the innate immune system responds first (i.e it is a “rapid reaction force”) with monocytes / macrophages, neutrophils, NK cells, and innate lymphoid cells (ILCs) recruited to the site of challenge to do most of the work of clearing the pathogen. Adaptive immune responses may follow in which tissue resident dendritic cells [41] instruct antigen-specific T cells and, indirectly B cells, both of which include cytokine-producing cytotoxic cells as well as memory cells. Key molecules in this identification/recognition process are major histocompatibility antigens, discovered by Dausset in 1954 [reviewed in ref. 42]. In evolutionary terms these refinements in adaptive immunity likely appeared about 100 mya. The mechanisms of action were partly elucidated in studies of skin graft rejection by Medawar and others [reviewed in ref. 43] which ultimately led to the notion of immune privilege (see below).

This textbook paradigm of how the immune response evolves after a single antigen challenge is predominantly based on studies in “frontier” tissues that border the external environment, such as skin, respiratory tract and gastrointestinal tract. These sites all have a rich microbiome with an extensive rapidly responding local immune cell population plus the potential for ready recruitment of bone marrow-derived innate immune cells through the vascular system. It is now realised that each tissue imprints its own stamp on the immune response, and indeed as Matzinger and Kamala have stated, the immune response is “primarily tailored to fit the tissue in which the immune response occurs” [44]. In one sense this statement might work as a definition of immune privilege.

## Is there an ocular immune system?

There is only an ocular immune system in as much as there is a lung immune system, a skin immune system or a skeleto-muscle immune system. It is likely that each tissue determines the quality and strength of the immune response [44]. Therefore, it is important to place the eye itself in an evolutionary context since it comprises several different tissue types.

### (a) Evolution of the eye

**Fig. 3:**
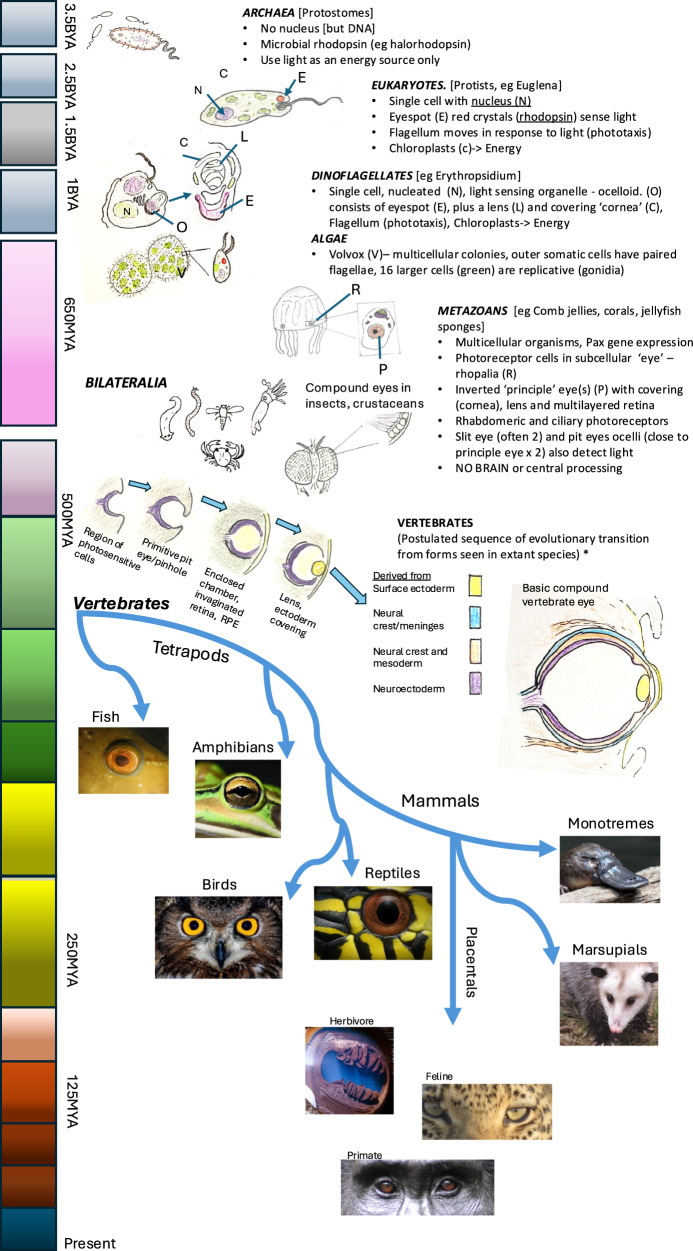
Evolution of life Forms with Photosensitive Organelles. Basic summary of the evolution of life forms with photosensitive organelles such as Euglena which aid, via phototaxis and diurnal rhythms, the emergence of multicellular animals and eventually bilateralia in which various light-gathering and receptive organs (‘eyes’) arise in forms of life in which there are two eyes which have connections to a central neural network (brain). Time scale on left is very approximate. [for an extensive consideration of the early evolution of photoreception, opsins and the evolution of the vertebrate eye, with reference to ciliary and rhabdomeric photoreceptors see refs. 98, 99].

Photoreceptors (rhabdomeric and ciliary) are thought to have arisen several times throughout evolution in vertebrates and invertebrates, but a camera-style eye likely made its appearance around 500 mya (soft tissues tend not to fossilise so dating is based on patterns in extant animal groups) [45]. Light-detecting crystals which form the primitive light-sensing organ, (the “eyespot”), found in some unicellular organisms close to the flagellum (such as Euglena) act to direct movement towards light (Fig. 3). In other organisms they may act to regulate circadian rhythms and seasonal variation in the photoperiod.

Primitive photoreceptors evolved through several iterations to diverge as two main eye types: the simple eye and the compound eye [46, 47]. The simple eye was based on the development of an eye cup (an invagination) with a pin hole caused by retrograde migration of the sheet of eyespot cells (Fig. 3), while the compound eye evolved from convex bulges (evaginations) in individual or groups of eyespot cells with multiple duplications [45]. Both arrangements allow detection of directionality of light which then makes it possible to have responses to that light. The co-evolution of specialised pigment cells behind the primitive photoreceptors acted to absorb excess light, which again helped in recognition of the spatial characteristics of the light. Both invagination and evagination allowed the development of specialised intervening transparent cells or matrix to form a primitive lens, which could contain protein, minerals or carbohydrates as long as it had a degree of transparency.

The vertebrate camera-style eye likely evolved from a type of simple eye but is more complex (based on a focussing lens and ciliary photoreceptors) [45, 46]. The photoreceptive component evolved from an outpouching (or evagination) of the central neural structure (tube), which folded in on itself to form an “eye-cup” (the invagination model) with the invaginating layer destined to form a three-layered neural retina. Ingressing surface ectoderm gives rise to the lens and the corneal epithelium. Neural crest cells arising from the junction of the neural tube and the ectoderm form most of the mesenchyme which, in the case of the eye, contributes to various ocular structures including the remainder of the cornea and the uveal tract (iris stroma, ciliary body stroma and choroid). Mesodermal cells give rise to vascular tissues [48] including the hyaloid vascular system and extraocular muscles [49].

In evolutionary terms therefore the vertebrate eye and its adnexa are composed of a number of tissues of different embryological (and therefore evolutionary) origins each of which will interact with the co-evolving immune system in a tissue-dependent way [50].

### (b) The ocular immune system

The ocular immune system has come to be considered unique in part because of the notion of immune privilege. During his studies on skin graft rejection, Medawar demonstrated that heterologous skin allografts placed in the anterior chamber (AC) of the eye were not rejected unlike allografts placed in the skin which were rejected [51]. These experiments and other work led him to propose that the anterior chamber (AC) of the eye was protected from the collateral damage of the immune / inflammatory response that allografts normally induce. Some years later Medawar’s post-doctoral fellow coined the term for this phenomenon “immune privilege” [52]. Medawar, meanwhile, noted that allograft survival in the AC was not 100%. Grafts were rejected if they connected with the vascularised iris tissue. On this basis, immune privilege was considered to be due to the lack of a tissue vasculature and a lymphatic drainage. Medawar proposed that this principle also explained the relative success rate of unmatched corneal grafts [discussed in refs. [53, 54]].

The concept that aqueous humour drained from the AC *via* the outflow system into the venous system on the surface of the limbus has strong homologies to the drainage of CSF in the subarachnoid space via the arachnoid villi to the dural venous sinuses, and has a long history [55]. Medawar also noted that allografts survived in the brain parenchyma (he also made reference to the testes) and proposed that immune privilege was unique to certain tissues. However, in more recent work, immune privilege and immunological tolerance generally, are viewed as properties of a wider range of tissues, some of which have well developed vasculatures, such as the gut [56]. From the above description of the evolution of the eye and its varied embryological tissue origins (surface ectoderm, neural ectoderm and neural crest derived mesenchyme), it would seem unlikely that mechanisms underlying a down-regulated immune response to an anterior chamber graft, applied to the retina. Instead, tissue barriers were proposed to underwrite CNS “immune privilege” since both the retina and the brain have highly developed and effective blood-tissue barriers with physiological roles in maintaining the unique cellular and extracellular milieu required for neural transmission [55] (Table 1). Indeed, in both the eye and the brain, two layers of vascular barrier exist (the blood aqueous barrier, BAB, and the blood retinal barrier, BRB, in the eye; the blood-CSF barrier, B-CSF, and the blood-brain barrier, BBB in the brain) [reviewed in ref. 50]. However, various degrees of blood-tissue barriers exist in all tissues, including the skin, the lung, the kidneys, and other tissues, and, as indicated above, in some of these tissues a measure of “immune privilege” is becoming more widely recognised [56].

**Table 1:** Moderators and mediators of the immune response in ocular tissues.

Mechanism	Cornea / Sclera	Lens / Vitreous	Uvea	Retina / Optic Nerve	references
Blood vessels	No	No	Yes + +	Yes+	[49–51]
Lymphatics	Inducible	No	controversial	“glymphatics”+ optic nerve meningeal	[61, 62, 65, 66]
Resident antigen presenting cells	Yes (dendritic cells and macrophages)	? (hyalocytes)	Yes (dendritic cells and macrophages)	No (microglia, perivascular macrophages)	[50, 70, 71]
Tissue immunosuppr-essive factors	Yes (TGFβ,FasL,CTLA-2 αMSH)	Yes		yes	[57]
Blood tissue barriers	Blood aqueous barrier (BAB)	BAB	BAB*; Outer Blood retinal barrier (BRB) **	Inner BRB ***, glia limitans	[49, 63]
Immune regulatory cells	Inducible	Inducible	Inducible	Inducible	[70, 81, 82, 86, 87]

^*^Blood aqueous barrier at iris, ciliary body.

**outer BRB at retinal pigment epithelium (RPE).

***inner BRB at retinal blood vessels.

Besides the cellular and vascular barriers there are non-cellular factors that likely contribute to the immune status of the ocular (and brain) environments. High concentrations of immunomodulatory molecules such as CTLA-2, TGFβ, CGRP,αMSH and more [reviewed in ref. 57] (see Table 1) secreted by barrier cells such as the retinal pigment epithelium (RPE) have been suggested as mediators of immune privilege, but much of the evidence comes from in vitro studies and whether this is significant in vivo is less clear. It is therefore likely that the immune response in most tissues is modified by the tissue barriers, but barriers in themselves do not prevent immune-mediated tissue damage or the invasion of pathogens. This is clearly illustrated by the fact that uveitis/retinitis and encephalitis are not uncommon conditions [58, 59]. Interestingly, recent evidence suggests the development of the BBB in the neonate (and presumably the BRB) are subject to control by the microbiome [60] inferring a role for the microbiome in the development of the CNS immune microenvironment.

Controversy remains over the question of lymphatic connections from the eye to the secondary lymphoid tissue, but it is important to differentiate between neural tissue (retina) and mesodermal/mesenchymal and ectodermal-derived tissues. The retina has no lymphatics. In addition, bona fide lymphatic vessels have not been empirically detected in any ocular tissues, including the uvea in mammals [61]. However, the eye in experimental mammals has a site-specific draining lymph node (submandibular node in mice) which likely communicates with ocular mucosal/conjunctival lymphatics since extirpation of this node prevents corneal allograft rejection [62] [reviewed in refs. 50, 63], suggesting that conjunctival lymphatic connections exist with non-retinal ocular tissues. In addition, Schlemm’s canal has been described as a lymphatic-like vessel [64] raising the possibility that this route may provide a connection between the intraocular space and lymphatic vessels in the conjunctiva.

Similar controversy exists concerning lymphatics in the CNS. Most studies describing CNS lymphatic connections have failed to differentiate between conventional lymphatics which are present in the dura mater and the lack of such lymphatics in the CNS parenchyma [reviewed in ref. [55]]. A CNS fluid transport system, termed the “glymphatic system” due to its close association with perivascular foot processes of glial cells contributes to the neurovascular unit (NVU). It has been proposed as a means by which brain interstitial fluid (ISF) can drain into meningeal lymphatics [65, 66]. The glymphatic system in effect comprises a potential space surrounding blood vessels in the brain parenchyma which expands and contracts in the sleep / wake cycle, thereby removing waste materials [reviewed in ref. 66]. Brain ISF is very limited but currently is considered to percolate through this space by capillary attraction, possibly bidirectionally depending on the pressure differential between the CNS arterial system and the CSF. Whether cells can also track through this route is unclear. However, similar “capillary channels” (conduits) supporting dendritic cell migration have been described in the stroma of lymph nodes [67].

Recent studies have suggested that the posterior vitreous cavity and the retina may have some form of “lymphatic communication” with meningeal lymphatics in the optic nerve sheath, which then drain to the deep cervical lymph nodes [68]. As described above lymphatics are present in meninges [55], but no vessels with the anatomical, ultrastructural or molecular markers of lymphatics have been described within the mammalian eye [61].

The lack of immunohistochemical evidence of MHC Class II expression, and, by inference the absence of dendritic cells, in ocular tissues such as the cornea has also been cited as further evidence for a deficient “afferent” arm of the ocular immune response [69] to explain immune privilege. However, later more extensive immunolabelling studies revealed networks of MHC Class II positive cells and macrophages within the cornea and uveal tract of the eye [reviewed in ref. 70] and meninges (pia, arachnoid and dura mater) of the brain [55, 71]. Importantly, the normal retina and brain parenchyma do not contain dendritic cells. They do however have specialised myeloid cells, namely yolk sac-derived microglia ( = CNS resident macrophages). The role of these cells is a house-keeping one (synaptic trimming) rather than as antigen presenting cells [72, 73]. Their physiological role appears to be immunoregulatory. However, they can act as accessory cells in immune-mediated inflammation [74]. In this sense therefore, absence of bona fide dendritic cells may contribute to the immunomodulatory environment of the retina and brain parenchyma. Low levels of MHC Class I as well as the presence of non-conventional MHC molecules in eye tissues [75] have also been suggested to contribute to immune privilege in the eye but the evidence is limited. However, there is some clinical support for what is described as MHC Class-I-opathies [76] but their relationship to ocular immune privilege remains unclear.

In the last 4 decades almost all components of the immune response have been implicated in some way to explain “immune privilege” in the eye (Table 1). These include absence of blood vessels and lymphatics, lack of antigen presenting cells, tissue factors regulating immunity, and tissue barriers [reviewed in refs. 57, 63, 77, 78]. Anti-microbial peptides have also been detected in the intraocular compartments [79]. However, immunomodulatory factors are present in many tissues, including the skin, lung and gut, and are not unique properties of ocular tissues.

It has also been suggested that local Tregs may contribute to immune privilege. Small numbers of circulating T cells, including Tregs, may traverse the fenestrated, sinus-like vessels of the uvea [reviewed in ref. 80] and ocular tissues, including the retina, may retain small numbers of T resident memory (Trm) cells and Tregs for prolonged times post-infection [81–84] but the normal healthy retina has few, if any, Tregs [81, 85]. More likely, Tregs are recruited to the eye in the resolution phase of inflammation as in any other tissue [86, 87].

An unexplained phenomenon which has occupied the pages of the ocular immune literature over many years is anterior chamber-associated immune deviation (ACAID) [88]. The term arises from the more general concept of immune deviation. The term “immune deviation” was coined in the early days of immunological discovery and described how an adjuvant-mediated delayed type hypersensitivity (DTH) skin reaction, similar to the tuberculin reaction, could be prevented in an antigen-specific manner by prior exposure to the antigen (in saline) in the skin [89]. Instead of the expected cell-mediated skin reaction (macrophages and T cells) a predominantly humoral (usually a non-complement fixing IgG2) antibody response is elicited to the second inoculation, despite the use of adjuvant. The immune response was thus considered to have “deviated” from a cellular to a humoral response (immune deviation). The same phenomenon occurs if the first inoculation of antigen (in saline) is intraocular rather than into the skin. This became known as anterior chamber-associated immune deviation (ACAID).

Skin-associated immune deviation was shown to be highly dependent on the dose and the physico-chemical nature of the antigen while ACAID was additionally dependent on the calibre of the cannulation needle used to inject Ag into the AC [90]. ACAID has been used experimentally for many years to study the ocular immune response [91, 92], but whether this phenomenon occurs as a natural response, for instance to microbial challenge, is not known. The relevance of immune deviation generally has declined as greater understanding of immune mechanisms and how they are regulated has accrued.

In the context of the ocular immune response, ACAID and immune privilege should not be confused. ACAID is a form of experimentally inducible immune tolerance which occurs in mice and rats in response to antigen in the absence of danger signals (such as those present in adjuvant) and can be induced by mucosal, subcutaneous, intravenous and intraocular administration of soluble antigen. Immune privilege, in contrast, describes how different tissues respond to antigen and depends on the nature of the tissue (see above). Neither inducible “immune deviation” or naturally occurring “immune privilege” are unique to the eye and both terms may be misleading in their mechanistic implications.

**Fig. 4:**
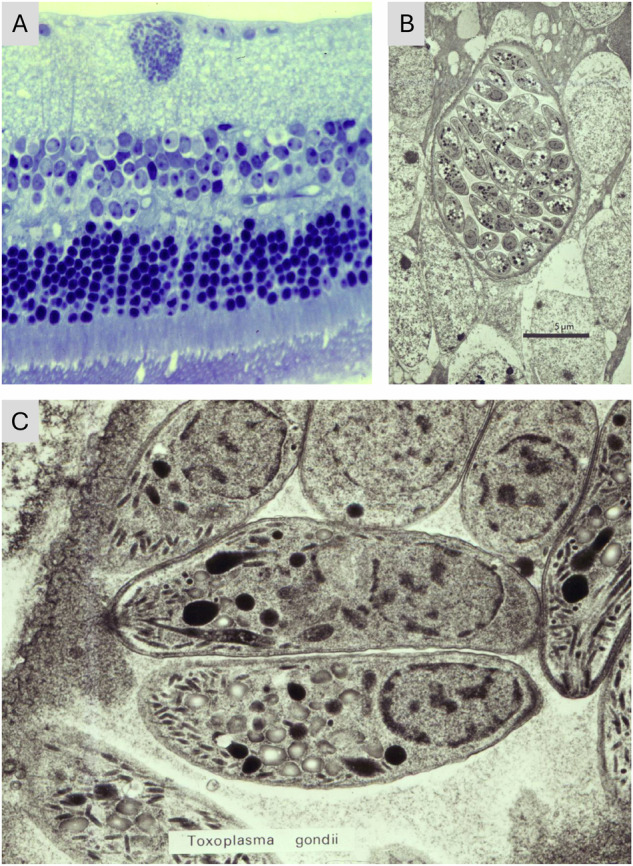
Histopathology of Toxoplasma Cysts in the Mouse Retina. **A** Light micrograph of mouse retina showing a Toxoplasma cyst residing in the inner retina with no obvious signs of an inflammatory/host response. **B** low power electron micrograph showing a Toxoplasma cyst containing many bradyzoites surrounded by host glial and neural cells of the inner nuclear layer. **C** high power electron micrograph of two bradyzoites. In some mice (not shown) in this model of congenital toxoplasmosis [100] there was marked inflammation in the retina [101].

Some pathogens do take advantage of the immune tolerant environment of the eye to evade detection. Pathogens which invade the intraocular compartment (ie breach the blood-aqueous barrier or the blood retinal barrier) may induce a self-limiting uveitis and enter a state of latency. For instance, in mice systemic CMV infection induces a short-lived anterior uveitis which subsides within ten days, but the virus persists in latent form long term in ocular tissues [93]. Toxoplasmosis is a world-wide systemic infection (1 in 3 of the world population have been infected). Toxoplasma gondii tissue cysts/oocysts (Fig. 4), ingested through poorly cooked meat or after contact with faecal material from cats (the final host), generate an explosion of tachyzoites when exposed to digestive enzymes and which can cross the blood retinal barrier and re-establish as bradyzooites, which reside latently in the retina [94]. Latency in this case is dependent on the host regulatory response [95] which, if it fails, may result in disease. Similarly, herpes simplex virus is maintained in latency within neurones by perineural IFNγ-secreting CD8 + T cells [96].

Whether latency develops depends not only on host factors but on microbial virulence factors which have still to be clarified. For instance, low grade anterior uveitis following cataract extraction may be due to sequestration of low-virulence microbes in a thickened posterior capsule (biofilm) which has developed mutualistic and variably controlled “disease tolerance” [33, 97].

In the final analysis, the downregulation of the immune response (“immune privilege”) in tissues such as the eye, brain, testes, hair follicle and others, is mediated centrally by regulatory T cells which provide protection, not only to the eye and brain but to many tissues as they return to homoeostasis after immune challenge. Interestingly this property of tissues can decline with age [81], thereby increasing the risk of diseases, such as CMV retinitis.

## Conclusion

The evolution of the ocular immune system can thus be seen as one aspect of the evolution of immunity itself. Host defence strategies date back to the beginning of life (LUCA) and immune systems co-evolved with all life forms. The immune system is thus shaped by two major drivers: the host and the microbiome. Innate immunity, emerging from primitive responses by unicellular organisms around 2.0-2.5bya, to a mechanism for maintaining homoeostasis of the host ecosystem by ~700,000 mya, is the default host response to challenge (danger theory) and all multicellular organisms attempt to restore homoeostasis through this process (discontinuity theory). Innate immunity has some level of immunological memory (trained immunity and tolerance) to re-challenge, but adaptive immunity, which evolved later in vertebrates, has taken this to a high level of specificity with degrees of variation depending on the nature of the antigen. All tissues, including ocular tissues, respond differently to any one antigen depending on their immunological armamentarium and the educative influence of the microbiome. No one tissue is privileged as such, but each tissue has a different capacity to prevent disruption and to restore homoeostasis. Each of the tissues or components of the eye, in part due to their various embryological origins, has a different capacity to modulate the immune response (exert “privilege”) with border tissues (cornea, sclera, uveal tract) less equipped to do so than neural retina.
